# Timing Embryo Preservation for a Patient with High-Risk Newly Diagnosed Acute Myeloid Leukemia

**DOI:** 10.1155/2018/9807047

**Published:** 2018-05-13

**Authors:** Rebecca Ye, Benjamin Tomlinson, Marcos de Lima, Ehsan Malek

**Affiliations:** ^1^Case Western Reserve University, Cleveland, OH, USA; ^2^Adult Hematologic Malignancies and Stem Cell Transplant Section, Seidman Cancer Center, University Hospitals Cleveland Medical Center, Cleveland, OH, USA

## Abstract

Great strides have been made in the treatment of acute myeloid leukemia (AML) resulting in increased number of survivors over all age groups, but especially in patients of reproductive age. Given the gonadotoxicity of high-dose induction chemotherapy and subsequent allogeneic stem cell transplant, it is paramount that fertility preservation options are discussed and explored at the time of diagnosis as fertility preservation has been associated with greater quality of life in survivors. Starting the conversation early is especially important for female patients given the time needed for all currently available fertility preservation techniques. Furthermore, due to a lack of current guidelines for the optimal timing of treatment, patients often encounter difficulties trying to balance life-saving treatment and fertility preservation. We present a case of female patient of reproductive age diagnosed with AML who opted for ovarian stimulation, oocyte retrieval, and subsequent IVF following a cycle of induction chemotherapy with satisfactory results for both embryo generation and disease treatment.

## 1. Introduction

Although previously incurable, acute myeloid leukemia (AML) is now curable in 35–40% of patients under 60 who undergo induction high-dose chemotherapy with or without allogeneic hematopoietic stem cell transplant (allo-HCT). In the U.S., 1 in every 509 women and 1 in every 415 men under the age of 49 will develop AML [1], and the increased survival of AML patients during their reproductive years lends itself to the discussion of fertility preservation. While there are existing recommendations for fertility preservation in cancer patients, the optimal timing and type of fertility preservation in patients with AML or acute lymphoblastic leukemia that requires urgent life-saving treatment remain largely unknown [2].

Despite available techniques, female survivors of acute leukemia have the lowest rates of postcancer pregnancy [3]. This can be attributed to the need for immediate chemotherapy in leukemia patients as well as specific limitations of each fertility preservation technique. Ovarian tissue cryopreservation and posttreatment transplant, the most common alternatives for cancer patients needing immediate chemotherapy, can be done for both restoration of fertility and natural hormonal production [4]. However, the presence of leukemic cells in cryopreserved tissue introduces the risk of relapse in patients otherwise enjoying remission and has been deemed unsafe [5, 6, 7]. Oocyte or embryo cryopreservation is another option for patients from whom mature oocytes can be collected; however, mature oocyte banking has a low success rates. While immature oocyte banking is slightly more successful, both of these techniques require time for oocyte harvest [5]. We present a case of a patient in menacme diagnosed with AML who opted for ovarian stimulation and subsequent *in vitro* fertilization (IVF) after one cycle of induction chemotherapy with satisfactory results, emphasizing the feasibility of this approach in cooperation with the standard timing of induction therapy and allo-HCT.

## 2. Case Presentation

A 26-year-old woman presented to her primary care physician with a two-month history of recurring sore throat, fever, and gum bleeding/persistent hematoma. Physical exam was significant for cervical lymphadenopathy. Complete blood cell count showed WBC of 1,800/L with 21% circulating blasts, platelets of 8,000/L, and Hb of 5.4 g/dL. Subsequent computerized tomography (CT) confirmed cervical lymphadenopathy. Bone marrow biopsy and aspiration revealed 37% myeloblasts with immunohistochemistry studies showing *KMT2A-MLLT3* rearrangement; cytogenetics were positive for t(9; 11) translocation. Immunophenotyping showed CD13^+^ CD33^+^ CD34^−^ blasts with two populations (one CD117^−^ and one CD11b^−^). Molecular studies were negative for other abnormalities. A diagnosis of AML (subtype M5) was made.

The patient received induction chemotherapy (7-day infusion of daunorubicin (90 mg/m^2^) and 3-day infusion of cytarabine (100 mg/m^2^)). A repeat bone marrow biopsy on day 14 showed regenerating marrow with no abnormal blasts by flow cytometry.

The patient expressed concerns about her future fertility following chemotherapy and spoke to a fertility specialist regarding the possibility of embryo cryopreservation. Given the patient's emergent need for chemotherapy, depot lupron was administered before the start of chemotherapy. Ovarian stimulation and egg retrieval necessitated a 9–14 day window and absolute neutrophil count (ANC) > 500 on day 1 of ovarian stimulation as well as ANC > 750 on the day of retrieval. The patient's egg harvest was successful and resulted in the cryopreservation of nine fertilized embryos.

Following egg retrieval, bone marrow biopsy at recovery of counts demonstrated first complete remission. The patient underwent consolidation chemotherapy (high-dose cytarabine (3 g/m^2^)) and subsequent allogeneic stem cell transplant (allo-HCT) with double umbilical cord blood transplant.

## 3. Discussion

Given the risks of premature ovarian failure and resultant infertility facing patients undergoing treatment for malignancy, it is important that they receive accurate information about all available options regarding fertility preservation. However, patients with malignancies requiring emergent treatment are unable to immediately undergo IVF due to the time needed for oocyte stimulation and retrieval. Previous guidelines have suggested that since the chemotherapy agents used for treatment of AML are not significantly gonadotoxic, fertility preservation measures are not necessary unless high-dose chemotherapy and/or bone marrow transplants are included in the treatment plan [6]. However, new emerging genetic markers (e.g., FLT3 mutation) that dictate the future need for allo-HCT are generally not available at the time of diagnosis, when fertility counseling for oocyte or embryo cryopreservation would be warranted. Furthermore, patients of childbearing age with newly diagnosed AML often wish to preserve oocytes or embryos before further chemotherapy exposure to minimize the chance of birth defects in offspring [8].

While several studies have recommended ovarian tissue cryopreservation as a speedy alternative to the waits imposed by oocyte and embryo cryopreservation, there exists the very real risk of malignant hematological cells in preserved tissue transferring disease back into the host. While the time between initial screening, tissue harvest, and actual transplant means that more sensitive screening techniques can potentially prevent transplantation of diseased tissue [4], a murine *in vitro* study demonstrated that disease transference from cryopreserved tissue is both possible and highly likely [9]. In a 2010 study, the majority of immune-deficient mice with transplanted ovarian tissue from ALL patients developed leukemic masses within six months. Further examination of the transplanted ovarian tissue showed no follicular development, negating the transplanted tissue's reproductive potential. Furthermore, while RT-PCR has shown to be a fairly sensitive method of disease detection via molecular markers, it is not infallible [4, 9].

By contrast, a 2012 study using ovarian tissue retrieved from patients in remission demonstrated no malignancy when the tissue was transplanted into immunocompromised mice. In this study, RT-PCR detected some disease-specific markers, they were found at levels close to the detection limit, and the study raised questions about the transformative potential of malignant cells as well as the viability of malignant cells in immunocompetent hosts following bone marrow transplant [7]. While the results of this study are promising, they serve to reinforce the uncertainty around cryopreserved ovarian tissue as a safe and viable option for fertility preservation in patients with ALL.

Embryo cryopreservation is the current gold standard for patients requiring gonadotoxic chemotherapy. However, given the ANC and time requirements for ovarian stimulation and oocyte retrieval, there are currently no guidelines on the optimal timing of these interventions. Studies in breast cancer patients have shown no significant difference in survival or recurrence if chemotherapy is delayed postsurgery for fertility preservation measures [10], but the current literature lacks any information about similar delays in patients with AML or other hematological malignancies.

This patient underwent a cycle of ovarian stimulation and oocyte retrieval after one course of 7 + 3 induction chemotherapy. While there exists the hypothetical chance of gonadotoxicity, there also exists significant heterogeneity in terms of the degree of gonadotoxicity for different drugs. We believe that this patient's initial chemotherapy should not dramatically increase her risk of infertility due to its short duration and use of cytotoxic drugs other than alkylating agents, which are known to be among the most gonadotoxic.

More importantly than any number of options, though, is ensuring that patients are aware of and counseled on posttreatment fertility issues. Despite the growing effectiveness of fertility preservation techniques, multiple studies have demonstrated that only about 48–60% of women of reproductive age who have undergone potentially gonadotoxic cancer treatment received counseling about fertility preservation [11–13]. Surprisingly, one of the strongest predictive factors for receiving information about fertility preservation was found to be male sex, even though fertility is commonly perceived as a primarily feminine issue [11]. One of the important components of survivorship is the resuming of a normal life and being able to have children is one of the possible parts of that life. Receiving specialized counseling about fertility preservation has been associated with less regret and greater quality of life for survivors [13]; however, it appears that few patients are exposed to this benefit.

This case demonstrates that ovarian stimulation and embryo cryopreservation are a viable option for patients undergoing chemotherapy and that it is possible to time ovarian stimulation between initial induction and consolidation chemotherapy for successful oocyte retrieval and fertilization. Furthermore, it also serves as a reminder of the importance of fertility preservation and counseling for patients of reproductive age—a service shown to improve quality of life posttreatment—but still has yet to become fully integrated as a part of patient education in patients of reproductive age.
